# Out of my real body: cognitive neuroscience meets eating disorders

**DOI:** 10.3389/fnhum.2014.00236

**Published:** 2014-05-06

**Authors:** Giuseppe Riva

**Affiliations:** ^1^Applied Technology for Neuro-Psychology Lab, Istituto Auxologico ItalianoMilan, Italy; ^2^Department of Psychology, Università Cattolica del Sacro CuoreMilan, Italy

**Keywords:** body image, eating disorders, cognitive neuroscience, image schema, body shame, allocentric lock, autobiographical memory, episodic memory

## Abstract

Clinical psychology is starting to explain eating disorders (ED) as the outcome of the interaction among cognitive, socio-emotional and interpersonal elements. In particular two influential models—the revised cognitive-interpersonal maintenance model and the transdiagnostic cognitive behavioral theory—identified possible key predisposing and maintaining factors. These models, even if very influential and able to provide clear suggestions for therapy, still are not able to provide answers to several critical questions: why do not all the individuals with obsessive compulsive features, anxious avoidance or with a dysfunctional scheme for self-evaluation develop an ED? What is the role of the body experience in the etiology of these disorders? In this paper we suggest that the path to a meaningful answer requires the integration of these models with the recent outcomes of cognitive neuroscience. First, our bodily representations are not just a way to map an external space but the main tool we use to generate meaning, organize our experience, and shape our social identity. In particular, we will argue that our bodily experience evolves over time by integrating six different representations of the body characterized by specific pathologies—body schema (phantom limb), spatial body (unilateral hemi-neglect), active body (alien hand syndrome), personal body (autoscopic phenomena), objectified body (xenomelia) and body image (body dysmorphia). Second, these representations include either schematic (allocentric) or perceptual (egocentric) contents that interact within the working memory of the individual through the alignment between the retrieved contents from long-term memory and the ongoing egocentric contents from perception. In this view EDs may be the outcome of an impairment in the ability of updating a negative body representation stored in autobiographical memory (allocentric) with real-time sensorimotor and proprioceptive data (egocentric).

## Introduction

Eating Disorders (EDs), as clinicians involved in clinical practice know well, are one of the most resistant and frustrating forms of psychopathology: patients rarely seek treatment voluntarily and their clinical behavior is usually characterized by denial and resistance.

This is particularly true for patients with Anorexia Nervosa (AN) who may associate denial with an active subversion of therapy (Vitousek et al., 1998). Often characterized by ambivalent feelings towards treatment (Williams and Reid, 2010), these patients may start to manipulate their weight, to dispose of food when unseen, or to increase their level of physical activity.

If we look at the definitions of Feeding and EDs presented in the Diagnostic and Statistical Manual of Mental Disorders, fifth edition (DSM V) we find that the clinical cores of these disturbances are a dysfunctional eating behavior, an excessive concern and distress about body shape or weight and, for AN only, the persistent lack of recognition of the seriousness of the current low body weight (APA, 2013).

In particular, various authors suggest that the excessive focus on the body hides the functional and egosyntonic nature of these disorders (Vitousek et al., 1998; Rich, 2006; Williams and Reid, 2010): ED patients manage their disturbances as both an identity and an illness. In other words, unlike other mental disturbances, EDs are also experienced positively by those who live with them (Serpell et al., 1999; Vanderlinden, 2008): many unhealthy eating behaviors are functional within the context of the patient’s belief system, and their benefits include feeling special, gaining a sense of control, and feeling protected. But the question remains, why is this so?

Even though the answer to this question is critical to explaining treatment resistance in ED, the debate remains open (Treasure et al., 2010; Kaye et al., 2013). In particular, the etiology of ED is a controversial topic. A first approach considers ED as the outcome of dysfunctions in the neuronal processes related to appetite and emotionality (Kaye et al., 2009, 2013). A second approach explains ED as the outcome of the interaction between cognitive, socio-emotional, and interpersonal elements (Schmidt and Treasure, 2006; Fairburn, 2008; Cooper and Fairburn, 2011; Treasure and Schmidt, 2013).

In their recent reviews, Kaye et al. (2009, 2013) support the first hypothesis. According to them, patients with AN and Bulimia Nervosa (BN) share a dysregulation in the anterior ventral striatal pathway that may create a vulnerability for dysregulated appetitive behaviors. If the high level of self-control in individuals with AN—produced by an exaggerated dorsal cognitive circuit functioning—allows them to inhibit appetite, this does not happen in BN patients, based on their limited ability to control their impulses.

However, several psychologists (Fairburn et al., 2003, 2009; Schmidt and Treasure, 2006; Cooper and Fairburn, 2011; Treasure and Schmidt, 2013) propose a more complex picture. Schmidt and Treasure, in their revised cognitive-interpersonal maintenance model of AN (Schmidt and Treasure, 2006; Treasure et al., 2012; Treasure and Schmidt, 2013), identify in obsessive-compulsive features and anxious avoidance (particularly of close relationships) the key predisposing factors. These factors, when associated with relevant precipitating factors—e.g., teasing, social comparison, stressful events—lead to the start and perpetuation of the disorder. Fairburn et al., in their transdiagnostic cognitive behavioral theory, suggest that EDs share a dysfunctional system for evaluating self-worth (Fairburn et al., 2003; Fairburn and Harrison, 2003). Then, one or more additional maintaining factors, specific for the different EDs, lead to the start and perpetuation of the disorder: core low self-esteem, over-perfectionism, mood intolerance, and interpersonal difficulties (Fairburn et al., 2003, 2009; Cooper and Fairburn, 2011).

These models, even if very influential and able to provide clear suggestions for therapy, still are not able to answer several critical questions: why do not all the individuals with either obsessive compulsive features or with a dysfunctional scheme for self-evaluation develop an ED? What is the role of the body experience in the etiology of these disorders?

While cognitive science has incorporated a lot of clinical aspects, clinicians generally are more reluctant to incorporate a cognitive perspective in their view. Yet cognitive science tells clinical science that many phenomena that are taken for granted, such as body perception, body experience, and the sense of self, eventually need further explanations.

In this paper we suggest that the path to a meaningful answer requires the integration of these clinical models with the outcomes of two new branches of cognitive sciences: Embodied Cognition and Social Neuroscience (Cacioppo et al., 2006; Ziemke, 2007; Bridgeman and Tseng, 2011; Wilson and Foglia, 2011; Davis and Markman, 2012; Koziol et al., 2012; Lakoff, 2012; Brugger et al., 2013). The Embodied Cognition approach underlines the central role of the body in influencing the mind. In other words, cognition is dependent upon features of the physical body of an agent. Moreover, the Social Neuroscience approach underlines the interactions between the subject’s perception of his own body in relation to others’ bodies and as influenced by normative standards.

## Understanding the body: the contribution of embodied cognition

We know that EDs refer to a range of problems characterized by abnormal eating behaviors and beliefs about body image and shape. Here we suggest that the starting point for understanding these causal processes is the link between our body and our mind as described by Embodied Cognition.

According to Embodied Cognition, the body influences the mind in three separate but related ways: (Wilson and Foglia, 2011)

- *Body as Regulator*: the body regulates cognitive activity over space and time, ensuring that cognition and action are tightly coordinated.
- *Body as Distributor*: the body distributes computational and representational load between neural and non-neural structures.
- *Body as Constraint*: the body constrains the nature and content of the representations processed by that agent’s cognitive system.

Specifically, the theoretical concept introduced by Embodied Cognition to explain this influence is one of “image schema” (Johnson, 2007). In the following pages we will detail and discuss this.

### The role of spatial representations in embodied cognition

According to Johnson (1987), “An image schema is a recurring dynamic pattern of our perceptual interactions and motor programs that gives coherence and structure to our experience” (p. xiv). Image schemas have been suggested as playing a critical developmental role, forming both the basis of early cognitive development, and possibly extending to all sensory-motor perceptual modalities (Mandler, 2004, 2012).

Let’s try to clarify this concept. The basic idea is quite simple: human beings from birth are able to recode selected aspects of incoming perceptual data into an image-schematic form, mapping spatial structure onto conceptual structure (Mandler, 2004, 2012).

For example, an infant is able to develop the concept of THING by identifying spatially coherent objects separate from the environment. Then, he or she is able to develop the concept of PATH—the image schema of a THING following a trajectory in space—by seeing different objects going from one place to another. In the same way the infant can create the concept of CONTAINER by seeing different THINGS performing a PATH in or out of another one.

This example (Mandler, 2008) underlines how image schemas include schematic information, related to the spatial positions and relations of objects. Apparently this content is the product of processing in the dorsal visual stream and includes the specification of the spatial relationships between the perceived objects (schematic component), making use of metrical visual information from an egocentric viewpoint (Milner and Goodale, 2008; Mandler, 2012).

As infants begin to manipulate objects and to move themselves within the environment, they start to enrich this spatially based representational system with perceptual information processed in the ventral visual stream and medial temporal lobe, embodying the enduring characteristics of objects and their spatial relations in an allocentric format (Byrne et al., 2007; Milner and Goodale, 2008; Mandler, 2012).

This information includes perceptual (mainly visual) information representing objects (e.g., WHEELS, SEATS) and their attributes (e.g., shape, contour, and size). Using the same building-block approach discussed before, a toddler can develop more complex concepts by joining together image schemas with perceptual representations. For example the toddler may define the concept of a CAR as a CONTAINER, with four WHEELS and internal SEATS that can perform a PATH. Mandler (2010) offers in his review a synthesis of the main studies supporting this view.

It is beyond the scope of this paper to summarize the theory and critical issues related to image schemas (for a broader view please refer to these two Special Issues: (a) “Modalities of Social Life: Roadmaps for an Embodied Social Psychology” published in 2009 in the *European Journal of Social Psychology*; and (b) “Embodied and Grounded Cognition” published in 2011 in the *Frontiers in Psychology*). In this context we will focus only on the consequences of this vision relevant to our discussion.

The main assumption, shared by many cognitive scientists, is that our conceptual system is the result of the interaction of a dual representation system (Galati et al., 2010): schematic (allocentric) and perceptual (egocentric):

- The egocentric reference frame is referred to the body of the subject and allows the location of objects relative to the body center: within an egocentric reference frame we represent an object relative to ourselves.
- The allocentric reference frame is instead referred to a space external to the subject: within an allocentric reference frame an object is represented independently of our own current relation to it.

In this view, the role of the egocentric representations is “pragmatic” (Jeannerod and Jacob, 2005): the representation of an object using egocentric coordinates is required for reaching and grasping. Instead, the role of allocentric representations is “semantic” (Jeannerod and Jacob, 2005): the representation of an object using allocentric coordinates is required for the visual awareness of its size, shape, and orientation.

A recent experiment using direct human neural activity recording from neurosurgical patients playing a virtual reality memory game provides a first, but important, evidence of the existence of image schema (Miller et al., 2013). In their study Miller et al. (2013) found that place-responsive cells (schematic) active during navigation were reactivated during the subsequent recall of navigation-related objects using language.

In the study, subjects were asked to find their way around a virtual world, delivering specific objects (e.g., a zucchini) to certain addresses in that world (e.g., a bakery store). At the same time, the researchers recorded the activity in the hippocampus corresponding to specific groups of place cells selectively firing off when the subject was in certain parts of the game map. Using these brain recordings, the researchers were able to develop a neural map that corresponded to the city’s layout in the hippocampus of the subject.

Next, the subjects were asked to recall, verbally, as many of the objects, in any order, they had delivered. Using the collected neural maps, the researchers were able to cross- reference each participant’s spatial memories as he/she accessed his/her episodic memories of the delivered items (e.g., the zucchini). The researchers found that when the subject named an item that was delivered to a store in a specific region of the map the place cell neurons associated with it reactivated before and during vocalization.

This important experimental result suggests—as predicted by image schema theory—that schematic and perceptual representations are integrated in situated conceptualizations that allows us to represent and interpret the situation we are experiencing (Barsalou, 2013). Once these situated conceptualizations are assembled, they are stored in memory. When cued later, they reinstate themselves through simulation within the respective modalities producing pattern completion inferences (Barsalou, 2003).

In sum, as suggested by image schema theory, recalling an episodic memory using language involves recovery of its spatial context.

## From the body to the others and back: the contribution of social neuroscience

One key characteristic of human beings is their sociality. But how does social interaction modify the brain and in particular its shaping of our bodily representations? Despite the influence social interactions have in shaping the different components of our experience of the body, this is an area that has traditionally been ignored by neuroscience (Brugger et al., 2013). In particular, neuroscience seems to forget that we are conscious of our own body through experience of both its physicality—we perceive our body as a physical object in the external world—and it subjectivity—we experience our body through different neural representations that are not related to its physical appearance (Moseley and Brugger, 2009; Legrand, 2010).

Put differently, body representations may be differentiated according to their first- and third-person characteristics. As explained by Zwicker et al. (2012): “By first-person, we mean those aspects of body awareness that are typically exclusively available to the owner of the body—the self. By third-person, we mean those aspects of body awareness and knowledge that are at least potentially available to any observer” (p. 19). And as shown by the phantom limb experience, first- and third-person representations of the body can be different. More, we do not need a body to experience it: we can experience a painful arm even after its amputation.

**Figure 1:**
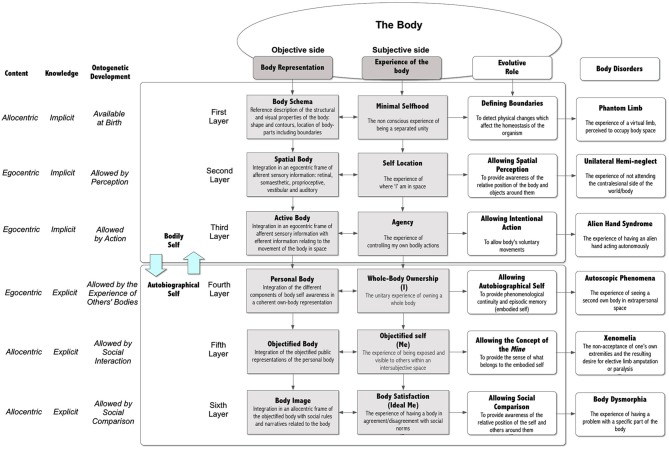
**Bodily self-consciousness and related disorders**.

However, the increasing interest of cognitive science and social psychology in the study of the experience of the body is providing a better picture of the process (Gallagher, 2005; Tsakiris et al., 2010; Blanke, 2012; Slaughter and Brownell, 2012). First, these studies support the idea that body representations play a central role in structuring cognition and the self. For this reason, the experience of the body is strictly connected to processes like cognitive development and autobiographical memory. Second, even though bodily self-consciousness is apparently experienced by the subject as a unitary experience, neuroimaging and neurological data suggest that it includes different experiential layers—minimal selfhood, self location, agency, whole body ownership, objectified self (*Me*) and body satisfaction (*Ideal Me*)—that are integrated in a coherent experience (Crossley, 2001; Vogeley and Fink, 2003; Blanke, 2012; Shilling, 2012; Pfeiffer et al., 2013). In the following pages we will suggest that these experiences are the outcome of different subrepresentations of the body—body schema, the spatial body, the active body, the personal body, the objectified body, and the body image—that are progressively integrated in a coherent and single representation through the subject’s interaction with the external and social world. Moreover, for each subrepresentation it is possible to identify one or more specific disorders that alter the bodily experience of the subject (Figure 1).

### The different dimensions of our representation of the body

As underlined by Rochat (2012), an innate body model (“body schema) allows at birth a primordial sense of embodied self-unity (“selfhood”) in infants, providing the capacity for separating our own embodied existence from the outside world: *I’m present in a body*. According to Damasio (1999) this non conscious state (*proto self*)—produced by a collection of neural patterns which are representative of the internal body’s state—allows the detection, moment by moment, of the physical changes which affect the homeostasis of the organism.

A more complex body representation develops slowly, through perception and action, over the first 3 or more years of life (Tsakiris, 2012). Specifically, this innate body model is supported by two online representations of the body (Ventre-Dominey et al., 2003; Giummarra et al., 2008; Tsakiris, 2010). In the first 6 months infants develop “the spatial body” (Self Location), produced by the integration in an egocentric frame of afferent sensory information (retinal, somesthetic, proprioceptive, vestibular, and auditory). This new body model allows the experience of where “I” am in space and that “I” perceive the world from there (Serino et al., 2013). As demonstrated by the classical experiment by Bahrick and Watson (1985) using contingent (live) and non contingent (pre-recorded) first-person videos (from the waist down) of the infant’s body, infants as young as 5 months of age are capable of discriminating between contingent and non contingent image of their own movements. Moreover, as demonstrated by different studies, the spatiotemporal information included in the spatial body allows infants to recognize different body parts, including faces and hands (Bonatti et al., 2002).

Then, in the second 6 months of life, infants start to develop “the active body”, produced by the integration of “the spatial body” with efferent information (Slaughter and Brownell, 2012) relating to the movement of the body in space (visual-proprioceptive integration). Within this representation toddlers do not perceive visual and proprioceptive body information separately, but as an integrated experience allowed by the perfect temporal correlation between proprioceptive (first-person) and perceptual (third-person) information (Zwicker et al., 2012).

From an experiential viewpoint this representation provides to the self the sense of agency, the sense that we control our own bodily actions. As demonstrated recently, even 4-month-old infants are sensitive to the relationship between the form and the motion of an agent and are able to integrate information about form and motion during action observation (Grossmann et al., 2013).

Counter-intuitively, however, these two body models are not enough to provide a sense of whole-body ownership, the unitary experience of owning a whole body (Tirassa et al., 2006; Tsakiris et al., 2006, 2010; Petkova et al., 2011a,b).

First, Tsakiris et al. (2010) recently identified the brain areas involved in body ownership and agency and found that they are qualitatively different experiences, recruiting distinct brain networks and triggered by different inputs. More, the brain areas involved in the processing of body parts are different from the ones integrating body parts in a full-body experience (Urgesi et al., 2007).

A second explanation is given by the progressive maturation of the specific brain regions involved in this experience: the temporoparietal junction (TPJ) and medial prefontal cortex. Using a fMRI study with infants between 15 and 30 months Lewis and Carmody found that the ability to reflect explicitly on self was related to the progressive maturation of left TPJ (Lewis and Carmody, 2008).

A third explanation is related to the lack of a whole-body representation in infants (Slaughter and Brownell, 2012): while young children are able to identify individual body parts by the end of the first year, they do not have a reflective awareness of the whole body until late in the second year of life, as indicated by personal pronoun usage, mirror self-recognition and representing their own and others’ behavior in pretense (Brownell et al., 2012). Concluding a review of their recent studies in this field, Brownell et al. underline (Brownell et al., 2012): “Our data suggest that children first become aware of their individual body parts in isolation from one another, then begin to represent their body as an obstacle in relation to other things in the world, then become able to consider their own body size explicitly, which is followed by representing how their body parts are arranged in relation to one another” (pp. 38–39):

In summary, the reflective experience of their own bodies in infants (“the personal body”) is the outcome of a developmental process happening between the 18th and 36th months of life: by paying attention to their own and others’ body actions, infants progressively integrate the different body locations into a whole body representation characterized by a specific size and shape (Brownell et al., 2007; Gilga and Southgate, 2012).

According to Damasio, the development of a whole-body experience allows the appearance of the autobiographical self (Damasio, 1999). In fact, this advanced self emerges only when—to quote the book’s title—self comes to mind, so that in key brain regions, the encoded experiences of past intersect with the representational maps of whole-body sensory experience. A key characteristics of autobiograhical self is episodic memory: a system which permits people to retrieve autobiographical memories characterized by temporal, spatial, and self-referential features—such as recalling having dinner with my friends yesterday (Souchay et al., 2013). Interestingly, only by the age of 3, children exhibit rudimentary episodic memory skills (Hayne and Imuta, 2011; Scarf et al., 2013): the ability to relate WHAT happened WHERE and WHEN.

Developmental studies underline the critical role of imitation in this process. In particular, through imitation infants develop two different representations: an integrated knowledge of their own body parts and actions and the map of this knowledge onto their knowledge of the body parts and actions of others (Jones and Yoshida, 2012). The final outcome of this second representation is “the objectified body”, an objectified public representation of our own body (Rochat, 2010): the body that others see, and more importantly, that they judge and evaluate. From the subjective side, the main experiential result is the “Objectified Self” (*Me*), the experience of the embodied self of being exposed and visible to others within an intersubjective space (Rochat and Zahavi, 2011). The development of “the objectified body” is the outcome of a long developmental process in which infants develop an embodied self not only through their own body, but also through how others perceive and represent it (Rochat, 2010; Rochat and Zahavi, 2011; Rochat, 2012).

As noted originally by psychoanalysis (e.g., Freud’s conflict between Id and Ego), the development of “the objectified body” is emotionally troubling for the individual. Rochat and Zahavi (2011), commenting on the ideas formulated by Merleau-Ponty on mirror self-experience, underline: “…the decisive and unsettling impact of mirror self-recognition is not that I succeed in identifying the mirror image as myself. Rather, what is at stake here is the realization that I exist in an intersubjective space. I am exposed and visible to others. When seeing myself in the mirror, I am seeing myself as others see me. I am confronted with the appearance I present to others. In fact, not only am I seeing myself as others see me, I am also seeing myself as if I was an other, i.e., I am adopting an alienating perspective on myself… The me I see in the mirror is distant and yet close, it is felt as another, and yet as myself… I cannot freely establish a distance and perspective on it, as I can with other objects. Indeed, I cannot get rid of my exteriority, my exposed surface” (p. 209).

Together with the concept of the *Me* children develop the concept of the *Mine*, the objectified sense of what belongs to the embodied self (Rochat, 2010, 2012). The main outcome of this process is reciprocity, a basic ingredient of human sociality (Rochat, 2010): children now consider their own objects alienable in the context of balanced social exchanges increasingly guided by principles of reciprocity and inequality aversion.

As noted by Rochat, “Reciprocity requires a concept of self that is enduring in a moral space made of consensual values and norms, a space in which the child becomes accountable and in which reputation starts to play a central role” (p. 743).

The emergence of the *Me* and of the *Mine*, underlines the critical role of culture in shaping our bodily experience: through it a variety of social inputs constructs and revises our own experience of the body. As suggested by psychologist Paul Schilder (1950): “…the child takes parts of the bodies of others into its own body-image. It also adopts in its own personality the attitude taken by others towards parts of their own bodies… There exists a deep community between one’s own body-image and the body-image of others. In the construction of the body-image there is a continual testing to discover what could be incorporated in the body” (pp. 137, 218).

This concept has recently been demonstrated experimentally (Tajadura-Jiménez et al., 2012): seeing someone else’s face being touched simultaneously as one’s own face produces changes both in the mental representation of one’s identity, and the perceived similarity of others. Although the influence of others’ bodies on our bodily experience is facilitated by vision and touch, real-life encounters with other people are not mandatory (Brugger et al., 2013).

Language and cultural practices, too, may have a direct impact on our experience of the body. In his classic paper “Techniques of the Body”, Mauss argued that cultures develop elaborate techniques of the body—highly developed body actions that embody the characteristics of a culture—which provide social actors with identities to conform to, rituals to perform, and other mundane activities to engage in (Mauss, 1973).

According to Shilling (2012), Western culture took another step forward, transforming the body into a symbolic project to be worked at and accomplished as part of the self-identity of the subject: “treating the body as a project…involves practical recognition of the significance of bodies as both personal resources and social symbols…Bodies become malleable entities to be shaped and honed by the hard work of their owners” (p. 7).

In this view the comparison/integration of “the objectified body” with an ideal societal body, expression of institutional norms and values, is a critical social and developmental process (Thompson, 1996; Thompson et al., 1999). Its main outcome is a new bodily representation—“body image”—that integrates the objectified representation of the personal body with the ideal societal body (the *Ideal Me*).

On one hand, by suggesting specific physical features the ideal societal body allows a formal adherence of the self to the rules and expectations of the society in which he or she lives. Specifically, individuals can decide to shape their physical bodies accordingly, to express agreement with embedded social norms. Plastic surgery is a classic example of a tool allowing individuals to reconstruct their bodies according to the social norms and expectations (Sarwer et al., 1998). On the other hand, the contents of “body image” shape body-related self-perceptions and self-attitudes, including thoughts, feelings, and behaviors. In particular, the main experiential outcome is “body satisfaction/dissatisfaction”, or how individuals feel about their bodies (Neumark-Sztainer, 2005).

Various studies suggest that body image, if present, is only rudimentary in children under 4 years (Smolak, 2012; Kerkez et al., 2013). If preschool girls (4-to-6 years old) demonstrate anti-fat bias and social comparison, body dissatisfaction and dieting are clearly evident only among elementary school-age girls (Smolak, 2012). Moreover, its development is strictly related to two different processes: the acquisition of advanced allocentric spatial memory abilities (Ribordy et al., 2013) and the emergence of autobiographical memory (Pathman et al., 2013).

### The interaction between the different components of our bodily representations

In the presented model the six representations are both implicit and explicit, and coded in a dual format (see Figure 1): egocentric (spatial body, active body, and personal body), allocentric (body schema, objectified body, and body image). Usually, the interaction between the egocentric and allocentric frames is limited (Longo et al., 2010): the experience of our moving body happens in a transient action-oriented egocentric perceptual frame, while our long-term memory of the body size is stored in an enduring allocentric environment–object representation (Burgess, 2006). Nevertheless, there is a permeability between them, allowing their interaction. Rossetti et al. (2005) underline that “several types of interaction can be observed between body image and body schema. Several techniques used to alleviate unilateral neglect produce a direct effect on body schema but nevertheless positively affect body image as well. In addition, the link demonstrated between the improvement of anosognosia and hemiplegia suggests that body schema and body image are tightly linked. It is concluded that dynamical and two-way dynamical interactions rather than simple static hierarchical links govern the relationships between body schema and body image” (p. 111).

But how do they interact? As suggested by Byrne et al. (2007) “Long-term spatial memory is modeled as attractor dynamics within medial temporal allocentric representations, and short-term memory is modeled as egocentric parietal representations driven by perception, retrieval, and imagery and modulated by directed attention. Both encoding and retrieval/imagery require translation between egocentric and allocentric representations, which are mediated by posterior parietal and retrosplenial areas and the use of head direction representations in Papez’s circuit” (p. 340).

Recently, Seidler et al. (2012) demonstrated the role played by working memory in two different types of motor skill learning—sensorimotor adaptation and motor sequence learning—confirming a critical involvement of this memory in the above interaction process. In general the interaction between egocentric perception and allocentric data happens through the episodic buffer of the working memory and involves all three of its components (Baddeley, 2012; Wen et al., 2013): verbal, spatial, and visuo-tactile.

Giudice et al. (2013) recently demonstrated that the processing of spatial representations in working memory is not influenced by its source—either perceptual or schematic. It is even possible to combine long-term memory data with perceptual images within an active spatial representation without influencing judgments of spatial relations.

A recent hypothesis is that these different representations can be integrated within the episodic buffer into an amodal (once formed, it no longer retains modality-specific information) spatial representational format shared by both perceptual and linguistic knowledge (Bryant, 1997; Loomis et al., 2007; Kelly and Avraamides, 2011; Wolbers et al., 2011; Baddeley, 2012). Both Bryant and Loomis identified this representational format in a three-dimensional coordinate system (Loomis et al., 2002, 2007; Bryant, 1997), available in the working memory, able to receive its contents both from multiple sensory input modalities (vision, tactile, etc.) and from multiple long-term memory contents (vision, language, etc.).

From an experiential viewpoint this amodal spatial representation may have two roles (Giudice et al., 2013): on one hand, it allows the control of action when the source stimulus is temporarily interrupted or removed; on the other hand it allows for the active imagining of layouts and the performing of mental transformations on them.

### A summary of the key points

After this long discussion we can summarize some key points that are relevant for a better understanding of EDs.

- *Our conceptual system is the result of the interaction of a dual representation system—schematic (allocentric) and perceptual (egocentric)*: these representations are integrated in situated conceptualizations that allow us to represent and interpret the situation we are experiencing. When cued later, for example through language, they reinstate themselves through simulations within the respective modalities, producing pattern completion inferences (Barsalou, 2003).
- *Our experience of the body is tightly integrated within a bodily self-consciousness that offers a single but layered experience of one’s body and oneself*: traditionally clinical research in EDs has studied separately the effects of the pathology on the different components of the experience of the body (i.e., body image and body schema). However, if we want to get the full picture we must remember that first, the bodily experiential layers are not two but six: minimal selfhood, self location, agency, whole-body ownership, objectified self, and body satisfaction; second, we do not experience these layers separately except in some neurological disorders; third, there is a natural, intermodal communication between them; and fourth, changes in one component can educate and inform other ones (Gallagher, 2005).
- *The different pathologies involving the different bodily representations suggest a critical role of the brain in the experience of the body*: these pathologies indicate that the brain does more than just detect and analyze sensory inputs; it may override and neglect sensory inputs or generate experience even in the absence of external inputs.
- *Our bodily experience evolves over time through the integration of different representations of the body, both allocentric and egocentric*: first, perception and action extend the minimal selfhood (body schema) existing at birth through two more online representations: “the spatial body”, produced by the integration in an egocentric frame of afferent sensory information, and “the active body”, produced by the integration of “the spatial body” with efferent information relating to the movement of the body in space. Then, through the maturation of the underlying neural networks and the progressive increase of mutual social exchanges, the embodied self is extended by further representations: “the personal body”, integrating the different body locations in a whole body representation; “the objectified body”, the integration of the objectified public representations of the personal body; the “body image”, integrating the objectified representation of the personal body with the ideal societal body.
- *The evolution of our bodily experience is related to perspective shifts, from allocentric to egocentric representations and vice versa*: in the ontogenetic development of our bodily self-consciousness it is possible to identify a clear evolutive path related to the ability of the subject in performing a more and more sophisticated shift from an egocentric to an allocentric perspective and vice versa: from the performing of spatial motor actions in the near space to their planning in the far space (from the here and now to there and future); from my body to the other one (from self to other); from the real-time experience of the body to the meaning of my body (from soma to psyche); from the meaning of my body to the social meaning of a body (from individual to social). A possible hypothesis, that we will discuss in the next pages, is that such fundamental reframing of the experience of the body and the self has a deep, if poorly understood, link with the etiology of EDs.

## From cognitive sciences to eating disorders

### An allocentric view of our bodies: the objectification theory

A popular socio-cultural model of EDs that clearly suggests the relevance of perspective shifts in the etiology of these disturbances is the “objectification theory”. Introduced by Fredrickson and Roberts (1997), this theory suggests that our culture imposes a specific orientational model—self-objectification—defining women’s behavioral and emotional responses (Calogero et al., 2010; Dakanalis et al., 2012; Riva et al., 2014). At its simplest, the objectification theory holds that (1) there exist an objectified societal ideal of beauty (within a particular culture) that is (2) transmitted via a variety of sociocultural channels. This ideal is then (3) internalized by individuals, so that (4) satisfaction (or dissatisfaction) with appearance will be a function of the extent to which individuals do (or do not) meet the ideal prescription (Tiggemann, 2011).

Specifically, women are taught to adopt a self-objectified view of themselves as bodies—focussing more on the *Me* and the *Ideal Me* than to the *I—*to meet Western cultural ideals of physical appearance and attractiveness (Fredrickson and Roberts, 1997; Daniel and Bridges, 2010). Using self-objectification to orient themselves in our society, women use body and appearance as their core basis of self-evaluation, with self-worth contingent on meeting the body shape ideals.

According to Fredrickson and Roberts (1997) repeated experiences of sexual objectification—when women are treated as bodies that exist for the use and pleasure of others—influence women to gradually adopt an observer’s perspective of their physical self; that is, they begin to treat themselves as an object to be looked at and evaluated on the basis of physical appearance (the *Me*). The self is so defined in terms of how the body appears to others.

The internalization of an observer’s perspective on one’s own body is labelled as “self-objectification” (Riva et al., 2014) and reduces a woman’s worth to her perception of her body’s semblance to cultural standards of attractiveness (Dakanalis and Riva, 2013b). Self-objectification is typically manifested as persistent body surveillance or habitual monitoring of the body’s outward appearance and is theorized to lead to a number of negative experiential consequences such as body shame, social physique anxiety, lack of awareness of internal bodily states, and decreased peak motivational states/flow experiences (Dakanalis et al., 2014a).

The link between self-objectification and EDs is supported by different correlational and longitudinal studies showing a direct link between ED symptomatology and self-objectification, body surveillance, and the internalization of sociocultural standards of beauty (Moradi and Huang, 2008).

A possible criticism to this vision is that males, who apparently are less prone to self-objectification, also experience EDs. However, different studies also underline the possible role of self-objectification in the etiology of male EDs (Dakanalis et al., 2012, 2013a,b, 2014b). Specifically in males, self-objectification is manifested as body surveillance (Dakanalis and Riva, 2013a). As in women, frequent body surveillance increases attention to disliked body parts, thereby encouraging the use of maladaptive eating and body shape regulation behaviors to modify the body (Calogero, 2009; Wooldridge and Lytle, 2012). More, body surveillance is strongly related to muscle dysmorphia (the belief in oneself appearing small and skinny, despite well-developed musculature), a male disturbance having symptomatic similarities with AN (Cafri et al., 2008; Murray et al., 2012).

Nevertheless, only a small subset of all the female and male subjects exposed to idealized body models develops clinically diagnosable EDs (Thompson et al., 1999). Why? A possible answer to this question is offered by the Allocentric Lock Hypothesis.

### The allocentric lock hypothesis

**Figure 2:**
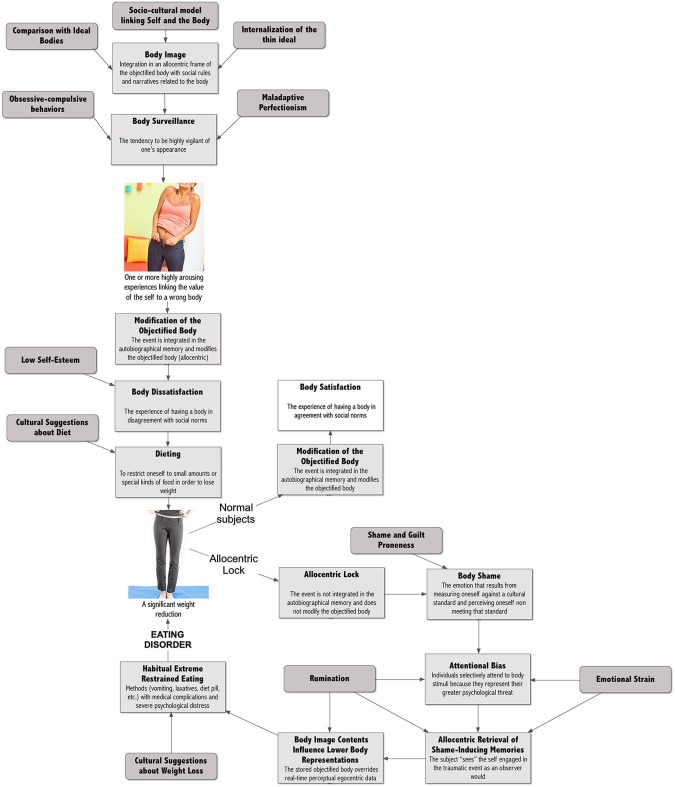
**From culture to eating disorders**.

The primary claim of the “*Allocentric Lock Hypothesis”* (Riva, 2007, 2011, 2012; Riva and Gaudio, 2012; Riva et al., 2012; Gaudio and Riva, 2013) is that individuals with EDs may be locked to an “objectified body” that is no longer updated by contrasting egocentric representations driven by perception. This claim is based on the following assumptions (see Figure 2) that are commented and justified in the next paragraphs:

- Subjects in their social interactions develop a specific “body image” that defines the meaning of our objectified body. The content of the body image is related to cultural standard. For example, this may include “fat phobia” (fear of becoming fat) in Western countries, or “weight phobia” (fear of becoming mature) in Asian countries (Lee, 1995).
- In Western countries the value of the objectified body is defined more by observable body attributes (e.g., “How do I look?”—allocentric perspective), rather than by privileged, or nonobservable body attributes (e.g., “What am I capable of?” “How do I feel?”—egocentric perspective).
- The endorsement and acceptance of appearance media ideals lead subjects to become aware of how their bodies looks and to evaluate themselves in terms of physical appearance. This evaluation becomes body surveillance, the tendency to be highly vigilant of one’s appearance (Fitzsimmons and Bardone-Cone, 2011).
- Subjects who experience one or more personal (e.g., “The new jeans were too tight”) or social (e.g., teasing) situations in which they fail to meet physical appearance standards, then update the objectified body accordingly (van den Berg et al., 2007): e.g., “My body is fat”. As demonstrated by different studies, including a recent meta-analysis (Menzel et al., 2010; Makinen et al., 2012), this produces body dissatisfaction, defined as displeasure with some aspect of one’s appearance (Cash and Pruzinski, 2004).
- Body dissatisfaction has a critical effect on eating behavior: subjects go on diet to improve the satisfaction with their body. According to the data collected by Ogden et al. from 8165 children and adolescents living in the United States (Ogden et al., 2008) about 62% of girls aged 12–19 report that they are trying to lose weight.
- Normally, after a successful diet, subjects experience a thinner body and again modify their objectified body accordingly (e.g., “I’m no more fat”). According to the Allocentric Lock theory, however, subjects with EDs are locked into their negative objectified body: its content cannot be updated even after a demanding diet and a significant weight loss.
- The impossibility of meeting societal standards transforms body dissatisfaction into body shame: the painful social emotion that can result from measuring oneself against a cultural standard and perceiving oneself as being judged and seen as inferior, defective or unattractive in the eyes of others (Pinto-Gouveia and Matos, 2011; Dakanalis et al., 2014a).
- Body shame usually has two behavioral effects: subjects either start more radical dieting behaviors—such as the use of diet pills and self-induced vomiting—or, at the opposite, decide to stop any form of food control and start “disinhibited” eating behaviors (Van Strien et al., 2009). In particular, as samurai committed seppuku to remove shame, subjects are ready to use the most extreme weight reduction techniques—fasting, vomiting, laxatives, etc.—to achieve the same result.
- A variety of studies show that shame experiences are also recorded in autobiographical memory, influencing body image and self-relevant beliefs, inattentional and emotional processing (Pinto-Gouveia and Matos, 2011). As demonstrated by numerous studies, in patients with EDs, the representation of the body included in the shame-inducing negative emotional event produces a priming effect on any body-related experience (Goldfein et al., 2000; Blechert et al., 2011): on one side, the tendency of our perception to be affected by our recurring thoughts produces an attentional bias on body related stimuli; on the other side, it draws the subject’s attention to previously stored body image stimuli biasing egocentric perceptual data and the interpretation of future self-relevant events.
- Specifically, shame experiences are retrieved in observer perspective, a way of remembering in which the subject “sees” the self engaged in the event as an observer would. During observer perspective patients shut down the neural circuitry in the insula switching off the real-time experience of the body: they are out of their body.

The next sections will deepen these points by detailing the possible causal processes leading to the development of the allocentric lock.

### A culture of dieters

As discussed before, according to the objectification theory in Western countries the value of the objectified body is defined more by observable body attributes (e.g., “How do I look?”—allocentric perspective), rather than by privileged, or nonobservable body attributes (e.g., “What am I capable of?” “How do I feel?”—egocentric perspective). Is this true? As demonstrated by a meta-analysis on 77 studies this approach defines the value of our objectified body (positive or negative) and the actions related to it (Grabe et al., 2008): its findings provide strong support for the relationships between media and internalization of the thin ideal as well as between media and women’s eating behaviors and beliefs.

The endorsement and acceptance of appearance media ideals lead subjects to become aware of how their bodies look and to evaluate themselves in terms of physical appearance. This evaluation becomes body surveillance, the tendency to be highly vigilant of one’s appearance (Fitzsimmons and Bardone-Cone, 2011). Why? According to McKinley and Hyde constant surveillance is needed to ensure that the subject complies with cultural body standards and avoids negative judgments (Fitzsimmons and Bardone-Cone, 2011). Seemingly, when one is concerned with weight/shape one would continue to survey the body with the hopes of monitoring and reducing those discrepancies.

**Figure 3:**
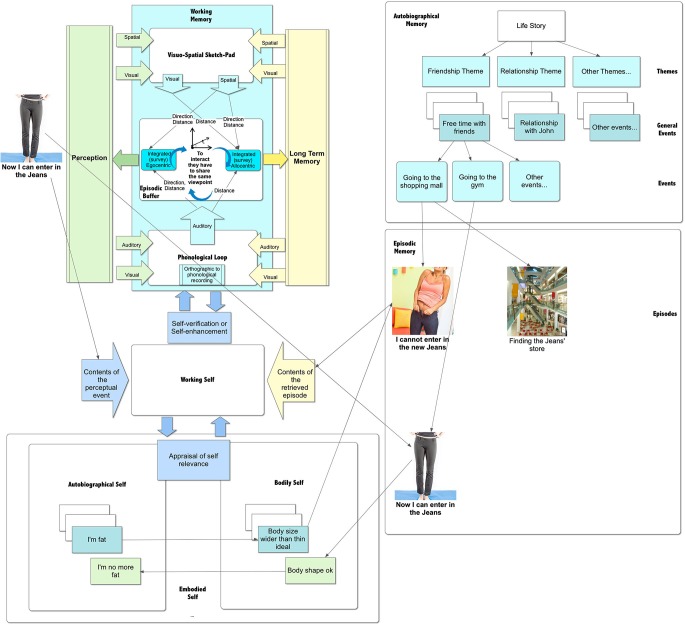
**Updating the contents of episodic memory**.

Subjects who experience one or more personal (e.g., “The new jeans were too tight”) or social (e.g., teasing) situations in which they fail to meet physical appearance standards, then update the objectified body accordingly (van den Berg et al., 2007): e.g., “My body is fat” (see Figure 3). As demonstrated by different studies, including a recent meta-analysis (Menzel et al., 2010; Makinen et al., 2012), this produces body dissatisfaction, defined as displeasure with some aspect of one’s appearance (Cash and Pruzinski, 2004). Three recent large community-based studies on normal subjects showed that the proportion of adolescent girls reporting body dissatisfaction is about one out of two (Makinen et al., 2012).

Cultural assumptions about weight include the belief that diets offer women relief from dissatisfaction with body size (Nell and Fredrickson, 1998). According to the data collected by Ogden et al. from 8165 children and adolescents living in the United States (Ogden et al., 2008) about 33% of girls aged 12–19 years are overweight. However, nearly double that percentage of girls (62%) report that they are trying to lose weight.

### Storing and updating a body related experience

As we have seen, one or more personal or social event in which the subject fails to meet physical appearance standards can modify the objectified body. But how does it happen?

Although our brain includes thousands of episodic memories, only some of them are included in autobiographical memories and are relevant to the self. According to Sutin and Robins (2008), “the critical defining feature for autobiographical memory is the importance of the information to one’s sense of self and one’s life history” (p. 1389).

Specifically, Conway (2005) suggested that autobiographical memory tries to construct and maintain a coherent self over time: when an autobiographical knowledge is retrieved, its content is continually evaluated by control processes to ensure that it is consistent with the individual’s current self-goals. According to Sutin and Robins (2008): the process involves the following (see Figure 3):

- *To evaluate if the memory is relevant to the self*: this evaluation, which may be implicit or explicit, compares the retrieved contents to the different representations of actual, ideal, and possible selves.
- *To evaluate if the memory is in agreement or not with the self*: this evaluation compares the self in the memory with the current self to verify the level of congruence (it is similar or different from the current self) and threat (it supports or reduces self-esteem).

In this view, the memory that the new pair of jeans were too tight, if related to the thin ideal, may be highly relevant to the self. However, what happens when an individual has a memory of herself that is congruent but threating for the self, like this one?

Swann et al. (2003) define this situation “a cognitive-affective crossfire”: the memory is cognitively congruent with the self but emotionally inconsistent with the need to maintain self-esteem. The cognitive-affective crossfire literature suggests that, in this situation, cognitive consistency tends to “win” over self-esteem enhancement. For example, subjects with low self-esteem prefer feedback congruent with their negative self-views to positive feedback inconsistent with their selves (Swann et al., 1987). More, a process of self-verification is activated to seek information that is consistent with the self-view (Swann et al., 2003). In sum, episodic memories of similar relevant experiences may produce an automatic, gradual updating of self-knowledge (Beike and Ransom, 2012). However, this process feeds into an implicit rather than explicit view of the self.

Another possibility is that the memory becomes available through a conscious decision process of *self-perception*: the individual, after examining the new event—the new pair of jeans were too tight—and considering the situation in which it occurred, comes to a conclusion about his/her self-knowledge—e.g., “My body is fat”.

The process also works in the opposite way. For example, after fitting into the pair of tight skinny jeans again, the individual can conclude that her body is now okay (see Figure 3).

This process is carried out through the interaction between the “working self” and the autobiographical memory knowledge base (Conway and Pleydell-Pearce, 2000; Conway, 2005). The *working self* is a temporary activation of current goals that constrain the search for elements to be bound up in the working memory. During the process of self-perception, the working self-retrieves the relevant memory (e.g., “My legs are fat”) from the autobiographical memory knowledge base and updates it with the perceptual data available in the working memory (e.g., “I now can fit into the Jeans, so I don’t have a fat body anymore”).

As discussed before, this process requires that the available representations share the same code. In other words, the schematic (allocentric) contents of the retrieved memory have to be translated into perceptual (egocentric) data. Recently, Burgess et al. (2001) and Byrne et al. (2007) proposed a model to explain this process. The allocentric representation (defining scene elements in terms of north, south east, west) is translated into an egocentric representation (defining scene elements in terms of left, right, ahead of the individual) by the retrosplenial cortex (RSC), with involvement of other cells: the place cells informing viewpoint location, the head-direction cells orienting the viewing direction, and the grid cells interpreting the self-motion signals. If, for some reason, this process is impaired, the subject is no longer able to update the representation of the body stored in the autobiographical memory. He/she is locked to it.

### The allocentric lock and its causes

The main tenet of the “*Allocentric Lock Hypothesis*” is that individuals EDs may be locked to an “objectified body” that is no longer updated by contrasting egocentric representations driven by perception (Riva, 2012; Riva and Gaudio, 2012).

There are various studies supporting this hypothesis. Foster et al., who assessed 59 obese women before, during, and after 48 weeks of weight loss treatment found that changes in the objectified body and in body dissatisfaction were not related to changes in weight (Foster et al., 1997). In a recent study, Guardia et al. (2013b) observed an altered representation of the body in a bariatric patient: the patient experienced a wider body even after a successful weight reduction (before: 125 Kg; after: 60 Kg). On one hand, this outcome is not uncommon and has also been found in about 30% of the patients experiencing laparoscopic adjustable gastric banding (Dakanalis et al., 2013c). On the other hand, Kaly et al. (2008) found a significant difference between what weight-loss clinicians and obese patients consider successful following bariatric surgery. As a general guideline, bariatric surgery is considered successful when 50% of excess weight is lost. However, most obese patients considered disappointing a 49% +/− 14% excess body weight loss, the gold standard for clinicians. Moreover, these results have some similarities with another recent work by Guardia et al. with anorectic patients (Guardia et al., 2012): in their study the patients experienced a wider body, and the magnitude of the overestimation was correlated with the size of the patient’s body prior to disease onset.

But why? Available research is not yet able to answer this question. Here, we suggest two possible causes of this lock: endogenous and exogenous. A possible endogenous cause is a structural and/or functional vulnerability of the brain areas involved in the egocentric/allocentric encoding process. Different recent neuroimaging studies revealed relevant brain alterations (Riva and Gaudio, 2012; Gaudio and Riva, 2013) in the key areas of both the core regions of the allocentric/egocentric frames and the core region of the egocentric frame (i.e., the precuneus and the inferior parietal lobe). Interestingly, such areas are affected in the early stages of AN (Gaudio et al., 2011) and are mainly related to the perceptive component of body image distortion (Gaudio and Quattrocchi, 2012). This possibility is also in line with the dysregulation in the anterior ventral striatal pathway suggested by Kaye et al. (2009, 2013).

A possible exogenous cause is an excessive level of stress. Different researchers recently suggested the influence of interpersonal problems (Hartmann et al., 2010), chronic stress, and post-traumatic stress on the onset of EDs (Troop et al., 1998; Sassaroli and Ruggiero, 2005; Rojo et al., 2006; Hepp et al., 2007; Lo Sauro et al., 2008). Recent research underlines the role of anxiety and stress in influencing the brain areas involved in the egocentric/allocentric transformation (Vyas et al., 2002; McLaughlin et al., 2007). The endogenous cholinergic tone in the dorsal hippocampus decreases with increases in anxiety and is associated with an increase in the serotonergic tone (File et al., 2000).

Further, a growing body of evidence has demonstrated that stress, and in particular chronic stress, can cause hippocampal damage (Vyas et al., 2002; McLaughlin et al., 2007), apparently through the excessive activity exerted by the amygdala on the hippocampus (Kim and Diamond, 2002). Specifically, chronic stress produces consistent and reversible changes within the dendritic arbors of CA3 hippocampal neurons (Conrad, 2006; McLaughlin et al., 2007), characterized by decreased dendritic length and reduced branch number. This process disrupts hypothalamic-pituitary-adrenal (HPA) axis activity, leading to dysregulated glucocorticoid release that, combined with hippocampal CA3 dendritic retraction, contributes to impaired spatial memory (Conrad, 2006).

Interestingly, a study demonstrated that the visual perspective for autobiographical memories was predicted by a gene × environment interaction between a polymorphism of the serotonin-transporter-linked promoter region (5-HTTLPR) and life stress exposure (Lemogne et al., 2009).

### The consequences of the allocentric lock on eating behavior

The impossibility of meeting societal standards transforms body dissatisfaction into body shame: the painful social emotion that can result from measuring oneself against a cultural standard and perceiving oneself as being judged and seen as inferior, defective or unattractive in the eyes of others (Pinto-Gouveia and Matos, 2011; Dakanalis et al., 2014a).

Theoretical accounts of shame hold that this emotion occurs from global attributions of failure (Nell and Fredrickson, 1998): failure is attributed to the self (e.g., “I am a bad person”), even if it is the result of a specific action of the self (e.g., “I did something bad) or just the intention of doing it (e.g., “I’m going to do something bad). An example of this effect is the “thought-shape fusion” effect found in many anorectic and bulimic patients (Kostopoulou et al., 2013): it occurs when the thought about eating a forbidden food (e.g., a high calorie food like dark chocolate) increases a person’s estimate of her weight/shape, elicits a perception of moral wrongdoing and makes her feel fat (i.e., “If I think about overeating or eating prohibited foods, then I think that I may have gained weight/I am immoral/I feel myself as fatter”).

In sum shame, including body shame, is accompanied by a sense of worthlessness and powerlessness that make it the more painful and “destructive” social emotion with a critical role in the development of different psychopathologies (Pinto-Gouveia and Matos, 2011). In fact, feelings of shame are more likely to motivate desire to hide or escape the shame-inducing situation, to sink into the floor and disappear (Cavalera and Pepe, 2014). As demonstrated by seppuku (*harakiri—*ritual suicide by cutting one’s own belly with a sword), individuals are ready even to lose their lives to remove shame: if you were *samurai* and lost your honor, harakiri was the only way to preserve it.

Body shame usually has two behavioral effects: subjects either start more radical dieting behaviors—such as the use of diet pills and self-induced vomiting—or, at the opposite, decide to stop any form of food control and start “disinhibited” eating behaviors (Van Strien et al., 2009). In particular, as samurai committed seppuku to remove shame, subjects are ready to use the most extreme weight reduction techniques—fasting, vomiting, laxatives, etc.—to achieve the same result.

The link between body shame and weight-disorder symptomatology has been verified by several authors. For example, Burney and Irwin found in a sample of 97 Australian women that shame associated with eating behavior was the strongest predictor of the severity of eating-disorder symptomatology (Burney and Irwin, 2000). Neumark-Sztainer et al. (2006), discussed the results of the Project EAT II (Eating Among Teens), a longitudinal study involving 2,516 ethnically and socioeconomically diverse adolescents. They report that, 5 years later, the use of unhealthful weight-control behaviors increased by six times the risk for binge eating with loss of control, three times the risk for being overweight, and two to five times the risk for extreme weight-control behaviors. A similar result was found by Stice et al. (2008a): in a different longitudinal study fasting was the best predictor for the future onset, 5 years later, of binge eating and BN.

### The consequences of the allocentric lock on self and the experience of the body

A variety of studies show that shame experiences are also recorded in autobiographical memory, influencing body image and self-relevant beliefs, inattentional and emotional processing (Pinto-Gouveia and Matos, 2011). How and Why?

Events of the personal history may be remembered using two different viewpoints (Robinson and Swanson, 1993; Rice and Rubin, 2009). The rememberer may “see” the event through his/her own eyes as in normal perception (*field mode*), or “see” the self-engaged in the event as an observer would (*observer mode*). The observer perspective is associated with a memory image focusing on the remembered self, whereas the field perspective focuses on the surrounding context (Frank and Gilovich, 1989).

Different studies suggest that, if healthy subjects tend to retrieve autobiographical memories in field perspective, many clinical samples preferentially remember personal events using an observer perspective. For example, Wells and Papageorgiou (1999) found that, in recalling anxiety-provoking social situations, individuals with social phobia and agoraphobia are more likely to take an observer perspective whereas control subjects are more likely to take a field perspective. A similar result was obtained by Bergouignan et al. (2008), in a sample of depressed patients, and by Osman et al. (2004) in a sample of patients with body dysmorphic disorder. Commenting these data Eich et al. suggest that shame-inducing negative emotional events are retrieved in observer perspective (Eich et al., 2012). In agreement with this, Bernstein et al. conclude (Berntsen et al., 2003): “increased access to the trauma memory and its role as a landmark leads to vivid and intrusive memories and over-inclusive classifications of other non-traumatic memories as related to the trauma. This is likely to generate a need by the traumatized person for distancing him- or herself from the phenomenological painful reliving and thus motivate the use of an observer perspective in remembering” (p. 690).

But what are the differences between field and observer modes of remembering?

A functional magnetic resonance imaging study demonstrated a different activation of the neural networks engaged with field versus observer memories for real-world events (Eich et al., 2009). The results revealed significant decreases in bilateral insula and left somatic motor activity during the recall of observer memories, suggesting a significant reduction in one’s cortical representations of the physical, embodied self. In other words, observer autobiographical memories have some similarities with the out-of-body experience (Blanke et al., 2004): “an experience in which a person seems to be awake and to see his body and the world from a location outside the physical body” (p. 243).

Commenting these results Eich et al. underline (Eich et al., 2012): “these findings have a number of interesting implications, both in the memory domain and beyond. For one, the data suggest that adopting an observer perspective is tantamount to a literal disembodiment at the neural level. That is, when we choose to relive past events from a perspective outside our body, we shut down the neural circuitry in the insula that is central for monitoring our bodies’ internal states. For another, research in social cognitive neuroscience has revealed that not only do we have a conceptual representation of “self” in the brain, but that it’s critical for how we process events in the external world. Building on this idea, our findings indicate that self-referential processing can also include a physical or somatic sense of self that is distinct from any conceptual or abstract self-representation” (p. 177).

According to Berntsen and Rubin (2006, 2007) the memory of a shame-inducing negative emotional event can become central to one’s life story and identity: through rumination, a recurring perseverative thought about the event (Connolly et al., 2014), the memory becomes highly interconnected with other types of autobiographical information in the cognitive networks of a person. This includes an understanding of the memory as a reference point for everyday inferences and for generating expectations, as a turning point in the life story and as a central component of identity (Berntsen and Rubin, 2006, 2007).

As demonstrated by numerous studies, in patients with EDs, the relevance given to the representation of the body included in the shame-inducing negative emotional event produces a priming effect on any body-related experience (Goldfein et al., 2000; Blechert et al., 2011).

On one side, the tendency of our perception to be affected by our recurring thoughts produces an attentional bias on body related stimuli. For example, the Stroop effects found for body/shape stimuli in AN patients (Dobson and Dozois, 2004), and the lower pressure detection threshold AN patients have on their abdomen (Keizer et al., 2012), apparently support this interpretation: anorectic individuals selectively attend to body stimuli because they represent their greater psychological threat (Dobson and Dozois, 2004).

On the other side, it draws the subject’s attention to the contents of the stored body image. More, the need for distancing him- or herself from a painful reliving motivate the use of a perspective outside our body. This switches off the neural circuitry used to monitor our bodies’ internal states (Eich et al., 2012) and biases egocentric perceptual data.

For example, in a recent study Guardia et al. (2013a). Evaluated spatial orientation constancy and the perception of body orientation in AN patients. They suggest that the poor perception of orientation can be related to lack of awareness of interoceptive signals (Guardia et al., 2013a). This result is in agreement with the study by Pollatos et al. (2008) assessing the perception of interoceptive sensitivity in AN. Their findings showed that interoceptive accuracy, as measured by a heart beat perception task, is reduced in AN patients (Pollatos et al., 2008).

More, Case et al. used a size–weight illusion (SWI; Berntsen and Rubin, 2007;—Subject experience the SWI when underestimating the weight of a larger object when compared to a smaller object of identical shape and weight) battery to compare visuo-haptic integration in anorectic and healthy females (Case et al., 2012). The authors found that the ability to discriminate weight did not differ between anorectic and control subjects. At the same time, subjects with anorexia showed a reduced SWI compared to controls. These results suggest that anorectics have a greater reliance on sensorimotor/proprioceptive memory compared to a reduced reliance on visual input in judgments of weight.

In a different study, Blechert et al. found that ED patients associate shape/weight concerns with the non-appearance-related domains of interpersonal relationships and achievement/performance (Blechert et al., 2011).

## Future directions

Even if the debate about the etiology of EDs is still open, clinical psychology is starting to explain EDs as the outcome of the interaction between cognitive, socio-emotional, and interpersonal elements. In particular, two influential models—the revised cognitive-interpersonal maintenance model and the transdiagnostic cognitive behavioral theory—identified possible key predisposing and maintaining factors (Schmidt and Treasure, 2006; Fairburn, 2008; Cooper and Fairburn, 2011; Treasure and Schmidt, 2013).

These models, even if very influential and able to provide clear suggestions for therapy, still are not able to answer to two critical questions: why do not all the individuals with either obsessive-compulsive features or with a dysfunctional scheme for self-evaluation develop an ED? What is the role of the body experience in the etiology of these disorders?

In this paper, we suggested that the path for a meaningful answer requires the integration of these models with the different outcomes of cognitive neuroscience. First, we underlined the critical role of the body in the development of our cognitive systems. In particular, our conceptual system is the result of the interaction of a dual-representation system (Galati et al., 2010)—schematic (allocentric) and perceptual (egocentric)—strictly related to the possibility of the body of interacting with the world: the representation of an object using egocentric coordinates is required for reaching and grasping; the representation of an object using allocentric coordinates is required for the visual awareness of its size, shape, and orientation.

Second, we suggested that our bodily experience evolves in time by integrating within the body image schema six different representations of the body—body schema, the spatial body, the active body, the personal body, the objectified body, and the body image—that are progressively developed through the subject’s interaction with the external and social world. Specifically, these bodily representations allow different experiences—minimal selfhood, self location, agency, whole body ownership, objectified self (*Me*) and body satisfaction (*Ideal Me*) that shape and enhance our bodily self-consciousness.

Third, Fredrickson and Roberts (1997), with their “objectification theory”, identified a specific cultural model that defines individuals’ behavioral and emotional responses in relation to their own body (Calogero et al., 2010; Dakanalis et al., 2012). Within this model, individuals are taught to adopt a self-objectified view of themselves as bodies to meet Western cultural ideals of physical appearance and attractiveness (Fredrickson and Roberts, 1997; Daniel and Bridges, 2010). Using self-objectification to orient themselves, women use body and appearance as their core basis of self-evaluation, with self-worth contingent on meeting the body shape ideals.

The link between self-objectification and EDs is supported by different studies showing a direct link between ED symptomatology and a self-objectified view of themselves. However, only a small subset of all the women exposed to idealized body models develops EDs. To explain this point, the “*Allocentric Lock Hypothesis”* (Riva, 2007, 2011, 2012; Riva and Gaudio, 2012; Riva et al., 2012; Cesa et al., 2013; Gaudio and Riva, 2013; Riva et al., 2013) suggested that EDs may be the outcome of a disturbance in the way the body is experienced and remembered: individuals with eating disorders may be locked to an “objectified body” that is no longer updated by contrasting egocentric representations driven by perception.

In our culture most women are dissatisfied about their body: one adolescent girl out of two reports body dissatisfaction (Makinen et al., 2012). More, cultural assumptions about weight include the belief that diets offer women relief from dissatisfaction with body size (Nell and Fredrickson, 1998). Normally, after a successful diet, subjects experience a thinner body and modify their objectified body accordingly (e.g., “I’m no more fat”) improving body satisfaction. According to the Allocentric Lock theory, however, subjects with eating disorders are locked into their negative objectified body: its content cannot updated even after a demanding diet and a significant weight loss.

The impossibility of meeting societal standards (e.g., I’m still fat) transforms body dissatisfaction in body shame: the painful social emotion that can result from measuring oneself against a cultural standard and perceiving oneself as being judged and seen as inferior, defective or unattractive in the eyes of others (Pinto-Gouveia and Matos, 2011; Dakanalis et al., 2014a).

Different studies show that shame experiences are recorded in autobiographical memory, influencing bodily self-consciousness and self-relevant beliefs, inattentional and emotional processing (Pinto-Gouveia and Matos, 2011). On one side, the tendency of perception to be affected by our recurring thoughts produces an attentional bias on body related stimuli: individuals selectively attend to body stimuli because they represent their greater psychological threat.

On the other side, it draws the subject’s attention to the contents of the shame experience. In particular, shame-inducing negative emotional events are retrieved in observer perspective, a way of remembering in which the subject “sees” the self engaged in the event as an observer would (Eich et al., 2012). As demonstrated by Eich et al., during observer perspective we shut down the neural circuitry in the insula that is central for monitoring our bodies’ internal states (Eich et al., 2009). If this experience, due to rumination, is repeated many times we can expect two effects: first, the real-time experience of the body is switched off—we are out of our own body; second, the real-time experience of the body is substituted by the contents of the objectified body stored in long-term memory.

Body shame is also accompanied by a sense of worthlessness and powerlessness that make it the more painful and “destructive” social emotion with a critical role in the development of different psychopathologies (Pinto-Gouveia and Matos, 2011). In particular, as samurai committed seppuku to remove shame, subjects are ready to use the most extreme weight reduction techniques—fasting, vomiting, laxatives, etc.—to achieve the same result. In sum, they develop some form of EDs.

Obviously, Allocentric Lock is still a hypothesis. So, in this final section, we present several avenues for future research on the Allocentric Lock hypothesis.

As discussed before, the hypothesis presented in this paper has multiple components that could be manipulated or assessed to test its validity. The first step to assess the processes presented in it would be to examine the presence of impairments of memory processes in EDs. Different studies suggest both impairment in autobiographical memory and working memory. For example, AN patients have problems in accessing emotional memories (Kova et al., 2011) and integrating both negative and positive emotional experiences (Nandrino et al., 2006) (autobiographical memory), and showed both hyperactivation in the parietal, and especially the temporal, lobe during a working memory task (Castro-Fornieles et al., 2010), as well as a compromised working memory performance under the influence of subliminal food images (Brooks et al., 2012) (working memory). However, further studies should assess the capacity of autobiographical and working memory in processing body-related content, and eventually, should identify the involved neural circuits.

Another way to explore the processes presented in the model would be to examine the ability of ED patients in manipulating egocentric and allocentric representations. Here too, even if some studies suggest impairment in this process (Smeets et al., 1999; Cooper and Mohr, 2012; Laghi et al., 2013), future studies should identify the extent of the impairment and its relation with both memory processes and the manipulation of bodily representations.

For example, a possible approach is to assess how egocentric and allocentric data interact within the different bodily representations discussed in the paper. Both Guardia et al. (2010, 2012) and Keizer et al. (2011, 2013) used interesting methodologies to reach this goal. Their data, even if preliminary, suggest that both conscious (i.e., the choice of a door-like aperture matching the perceived size of the body) and unconscious (i.e., the adaptive postural changes required to enter a door-like aperture) egocentric body-related judgments are impaired in AN (Guardia et al., 2012; Keizer et al., 2013). In both studies, AN patients significantly overestimated the size of their real body. Further, in the study by Guardia et al. (2012), the overestimation was positively correlated with the body weight prior to disease onset, as predicted by the Allocentric Lock hypothesis.

To more fully test the predictions made by the hypothesis, a further approach is the use of neuroimaging studies to identify and evaluate the neural circuits involved in the bodily experience of patients with EDs. For example, we have seen that the allocentric lock has many similarities with out-of-body experiences. Future researches may evaluate possible overlaps between the neural circuits involved in both processes. A preliminary study by McAdams and Krawczyk (2011) comparing AN patients with normal controls found an impairment in the neural processing of social attribution in the clinical sample. Specifically, they found a reduced activation in the right TPJ, a key neural locus for the etiology of autoscopic phenomena (Blanke et al., 2005).

It is also interesting to underline that the presented hypothesis fits well with both the existing etiological models of EDs. On one side, the allocentric lock produces a dysfunctional system for evaluating self-worth in line with the assumptions of the transdiagnostic cognitive behavioral theory (Fairburn and Harrison, 2003; Fairburn et al., 2003). On the other side it suggests a critical role of stress and anxiety related issues that fit well with the revised cognitive-interpersonal maintenance model of AN (Schmidt and Treasure, 2006; Treasure et al., 2012; Treasure and Schmidt, 2013).

Nevertheless, as for any new approach, much more research is needed before the proposed vision can be retained or discarded. However, it provides a possible explanation of EDs that addresses the complex etiology of these disturbances by including sociocultural and biological factors. Further, it suggests a clear link between EDs, the experience of the body, autobiographical and working memory, and the spatial reference frame brain areas, which may be helpful to enhance the therapeutic options for these disturbances.

First, a possible approach to counter the negative contents of the objectified self memory is the use of cognitive dissonance-based interventions (Stice et al., 2001, 2013; Perez et al., 2010). These interventions, used to prevent EDs, aim to induce cognitive dissonance with respect to standard of female beauty using small group activities and homework assignments in which individuals speak and act against the thin-ideal. As demonstrated by a meta-analysis (Stice et al., 2008b) and different controlled trials, this approach is able to reduce the risk for obesity and EDs onset with some effects persisting through 3-year follow-up.

Another possible approach to counter the negative contents of the objectified self memory is the competitive memory training—COMET (Korrelboom et al., 2009) for improving low self esteem in individuals with EDs. Starting from Brewin’s notion of a “memory retrieval competition” in long-term memory (Brewin, 2006) between the different components of the the same concept, this approach stimulate to retrieve and attend to positive autobiographical memories that are incompatible with low self-esteem, by using self-esteem promoting imagery, self-verbalizations, facial and bodily expression, and music. Tested in a controlled trial with 53 patients with EDs, this approach allowed a 27% higher clinical change than a traditional EDs treatment (Korrelboom et al., 2009).

Finally, the use of virtual reality (VR), a syntethic egocentric experience, to modify the experience of the body in eating disordered patient is an another emerging approach (Ferrer-Garcia et al., 2013). Ferrer-García and Gutiérrez-Maldonado conclude their recent review about the use of VR for the treatment of body image in EDs with the following words (Ferrer-García and Gutiérrez-Maldonado, 2012): “Several conclusions can be drawn from reviewed studies. VR-based therapies seem to be especially suitable for improving body image both in ED patients and in subclinical samples… All these studies showed significantly greater improvement in measures related with body image when the VR component was added” (p. 9).

In conclusion, we know the power of understanding etiology in the search for effective interventions: treatment is best accomplished when we know the causes of a disorder. For this reason, any step, even if partial, towards a better understanding of EDs may be useful for identifying new and better treatment options.

## Conflict of interest statement

The authors declare that the research was conducted in the absence of any commercial or financial relationships that could be construed as a potential conflict of interest.
